# The role of income and psychological distress in the relationship between work loss and smoking cessation: Findings from three International Tobacco Control (ITC) Europe countries

**DOI:** 10.18332/tpc/113092

**Published:** 2019-11-19

**Authors:** Karin Hummel, Bas van den Putte, Ute Mons, Marc C. Willemsen, Geoffrey T. Fong, Raphaël Andler, Hein de Vries, Gera E. Nagelhout

**Affiliations:** 1 Department of Health Promotion, Maastricht University (CAPHRI), Maastricht, Netherlands; 2 Department of Communication, University of Amsterdam (ASCoR), Amsterdam, Netherlands; 3 Cancer Prevention Unit & WHO Collaborating Center for Tobacco Control, German Cancer Research Center (DKFZ), Heidelberg, Germany; 4 Trimbos Institute, Netherlands Institute for Mental Health and Addiction, Utrecht, Netherlands; 5 Department of Psychology, School of Public Health and Health Systems, University of Waterloo, Waterloo, Canada; 6 Ontario Institute for Cancer Research, Toronto, Canada; 7 Santé Publique France, the National Public Health Agency, Saint-Maurice, France; 8 Department of Family Medicine, Maastricht University (CAPHRI), Maastricht, Netherlands; 9 IVO Research Institute, The Hague, Netherlands

**Keywords:** survey, unemployment, cessation, income

## Abstract

**INTRODUCTION:**

The relationship between work loss and smoking has not been studied extensively, and underlying factors are often not examined. The aim of this study was to test two hypotheses. First, work loss is associated with greater intention to quit and more likelihood of smoking cessation, and this relationship is moderated by a decrease in income. Second, work loss is associated with lower quit intention and lower rates of smoking cessation, and this relationship is moderated by an increase in psychological distress.

**METHODS:**

We used pooled data from three countries participating in the ITC Project: France, Germany and the Netherlands (n=2712). We measured unemployment, income and psychological distress at two consecutive survey waves, and calculated changes between survey waves. We first conducted multiple logistic regression analyses to examine the association between work loss and smoking cessation behavior. Next, we added income decrease and psychological distress increase to the models. Finally, we added interaction terms of work loss by income decrease and work loss by distress increase to the model.

**RESULTS:**

Work loss was not associated with quit intention, quit attempts, and quit success. When income decrease and psychological distress increase were added to the model, we found a positive association between distress increase and quit attempts. The interactions, however, were not statistically significant.

**CONCLUSIONS:**

Our results indicate that smokers who become unemployed and face a decrease in income are not less likely to quit smoking than smokers who are employed.

## INTRODUCTION

Previous studies have shown that people with a low socioeconomic status are more likely to smoke, be more addicted, and less likely to quit; these studies mainly focused on smokers’ income and educational level. Existing studies have given mixed results on the relationship between unemployment and smoking. Work loss is an important event in one’s life that can influence various behaviors such as smoking^1,2^. Several studies have investigated the relationship of work loss with income and psychological distress, while others examined the relationship of income and psychological distress with smoking^1,3-5^. Results show that work loss is often associated with an increase in psychological distress^3,6^ and related to increase in smoking^1,4,5^. To the best of our knowledge, only one study, conducted in the US, has examined the role of both income and psychological distress as factors in the relationship between work loss and smoking, and the authors concluded that only psychological distress played a role^1^. Specifically, work loss was associated with an increase in psychological distress, and this increase in psychological distress was associated with increased smoking. The aim of the present study was to examine how work loss affects smoking cessation among smokers from three European countries: France, Germany and the Netherlands.

Two competing hypotheses were tested in the present study. First, work loss is associated with greater intention to quit and higher probability of smoking cessation, but moderated by a decrease in income. Second, work loss is associated with lower quit intention and lower probability of smoking cessation, but moderated by an increase in psychological distress levels.

## METHODS

### Design and sample

We used data from the International Tobacco Control (ITC) surveys in three countries: France, Germany and the Netherlands^7,8^. Each of the ITC surveys used a longitudinal cohort design among nationally representative samples of smokers. We used two consecutive survey waves from each country: France 2008 and 2012; Germany 2009 and 2011; and the Netherlands 2009 and 2010. More detailed information about each country’s methodology can be found elsewhere^7-10^. For the current study, we included respondents who participated in both survey waves and who reported being a smoker in the first survey wave; smokers are defined as those respondents who had smoked at least 100 cigarettes in their lifetime and were currently smoking at least monthly. We pooled the data from the three countries. This resulted in a total of 2712 smokers (1070 from France, 547 from Germany, and 1095 from the Netherlands; the sample is described in Supplementary Table S1).

### Measurements

Unemployment was measured by asking the participants: ‘Are you currently employed outside the home (yes/no)?’. We defined work loss as having a work at baseline and not having a work at follow-up. Income was measured differently in the three countries. Respondents from France and Germany reported their monthly net household income (11 categories in France, 10 in Germany), while respondents from the Netherlands reported their monthly gross household income (13 categories). Income decrease was defined as a decrease of at least one category between survey waves; the reference group were respondents who experienced no change in income. Psychological distress was measured with a short version of the Perceived Stress Scale (PSS)^11^. We used the mean of the coding of responses to a set of four questions that could be answered on a 5-point Likert scale (1 = ‘very often’ to 5 = ‘never’). For example: ‘How often in the last 6 months have you felt that difficulties were piling up so high that you could not overcome them?’, Cronbach’s alpha for this scale was 0.68. Distress increase was defined as an increase of mean value between survey waves, with respondents who had no change in distress as the reference group. Quit intention was measured by asking: ‘Are you planning to quit smoking…’ on a 4-point scale ranging from 1 = ‘within the next month’ to 4 = ‘or are you not planning to quit?’^12^. This variable was dichotomized into whether smokers had the intention to quit within the next 6 months or not. Quit attempt was measured by asking: ‘Have you made any attempts to stop smoking since the last survey (yes/no)?’. Among smokers who had made a quit attempt between surveys, quit success was measured at the second survey wave by asking respondents about their smoking status, with successful quitters being those who indicated that they were not currently smoking. Age, sex, country of residence, the level of completed education, whether respondents lived with a partner who smokes, and whether respondents have friends who smoke were included as covariates. The surveys were approved by the Research Ethics Board of the University of Waterloo.

### Statistical analyses

We conducted three separate multiple logistic regression analyses: 1) quit intention, 2) quit attempts, and 3) quit success, as dependent variables. Age, gender, country of residence, education, living with a partner who smokes, having friends who smoke, and work loss were independent variables. Furthermore, we tested with multiple logistic regression analyses whether work loss was associated with income decrease and distress increase. Respondents had the opportunity to refuse to answer the income question. These respondents (in total 962, from all three countries) were excluded from the regression analyses. All statistical estimates and tests presented were weighted for sex and age. We calculated the degree of multicollinearity, and this was low with VIF values ranging 1.00–1.07^13,14^.

## RESULTS

### Sample description

Between survey waves, 23.8% experienced an income decrease and 36.6% an increase in psychological distress. A total of 8.5% smokers lost their work between survey waves. Among those, 33.7% experienced an income decrease and 39.2% an increase in psychological distress.

The drop-out rate between survey waves was 40%. Across countries, respondents who were young, male, and with a moderate educational background were more likely to drop out. Respondents who refused to answer the income question had lower quit intention (17% intended to quit within the next 6 months) than respondents who answered the income question (26%).

### Relationship between work loss and smoking cessation

Work loss between survey waves was associated with a decrease in income (OR=1.54, p<0.05), but not with an increase in psychological distress (OR=1.12, p=0.52). Work loss was not associated with any of the quit-related outcome variables (Table 1). When income decrease and distress increase were added to the model, the only significant result was that an increase in psychological distress was associated with higher odds to make a quit attempt (OR=1.41, p<0.01). The other associations in Table 1 were not significant.

**Table 1:** Logistic regression analyses for quit intention, quit attempt and quit success

	*Intention to quit within 6 months (yes/no) OR*	*Quit attempt (yes/no)*	*Quit success (yes/no)*
	*OR (95% CI)*	*OR (95% CI)*	*OR (95% CI)*
**Step 1**			
Work loss	1.16 (0.68–1.97)	0.80 (0.53–1.21)	1.49 (0.75–2.95)
**Step 2**			
Work loss	1.16 (0.68–1.98)	0.79 (0.53–1.20)	1.50 (0.75–2.99)
Income decrease	0.91 (0.66–1.26)	0.87 (0.68–1.11)	0.79 (0.52–1.23)
Psychological distress increase	1.26 (0.95–1.67)	1.41 (1.14–1.75)**	0.90 (0.63–1.29)
**Step 3**			
Work loss	1.70 (0.81–3.54)	0.77 (0.40–1.41)	1.37 (0.42–4.49)
Income decrease	0.96 (0.68–1.35)	0.92 (0.71–1.19)	0.80 (0.51–1.24)
Psychological distress increase	1.31 (0.97–1.76)	1.35 (1.07–1.68)**	0.89 (0.61–1.29)
Work loss* income decrease	0.56 (0.18–1.81)	0.50 (0.19–1.29)	1.05 (0.18–6.11)
Work loss* psychological distress increase	0.59 (0.19–1.84)	1.83 (0.78–4.31)	1.17 (0.27–5.05)

All analyses are adjusted for age, gender, country of residence, education, living with a partner who smokes, and having friends who smoke.

* p<0.05.

** p<0.01.

We performed sensitivity analyses in which we included income decrease, psychological distress increase and the interaction terms separately into the models. None of the results was statistically significant.

## DISCUSSION

The aim of the current study was to examine the relationship between becoming unemployed and smoking cessation, and to study the role of income and psychological distress as factors in this relationship. Previous research focused on either the relationship of unemployment and income/psychological distress, or on the relationship between income/psychological distress and smoking behaviors. Our results show that income decrease does not moderate the relationship between work loss and smoking cessation, thus, rejecting the first hypothesis. In contrast to our second hypothesis, we found that an increase in psychological distress was associated with a higher probability of a quit attempt, but the interaction with work loss was not significant. However, it should be noted that our hypothesis that work loss would be related to an increase in psychological distress was based on studies that examined involuntary work loss while it is possible that voluntary work loss might lead to a decrease in psychological distress. We did not have a measure of whether work loss was voluntary or not in our study.

Similar to our findings, one study has found an association between becoming unemployed and a reduction in income, and no relationship between income decrease and smoking^1^. The authors gave as a possible explanation that smokers may reduce their smoking or switch to cheaper tobacco products when their income decreases, but not quit smoking.

In contrast to our findings, previous research has shown that work loss was associated with an increase in psychological distress^1,3-5,15^. Furthermore, we found an association between an increase in psychological distress and making a quit attempt, which is contradictory to our second hypothesis. A possible explanation is reversed causality, which we could not test in our study because distress change as well as quit attempts were measured during the same period. Therefore, it is possible that smokers had tried to quit in the previous year and that this attempt increased their level of distress. Future research should examine the relationship between unemployment, psychological distress and smoking behaviors, using longitudinal data.

Our results indicate that a decrease in income and an increase in psychological distress do not play a major role as factors in the relationship between work loss and smoking cessation. Based on our findings, there is little reason to focus smoking cessation interventions specifically on smokers who have become unemployed.

### Limitations

There are some limitations in this study. First, we used self-reports with a high number of non-responses for income. Second, we had a relatively high number of respondents who dropped out between survey waves. Third, there were some methodological differences between the participating countries, especially the time between the survey waves and the data collection mode. Also, income was measured differently in the three countries. Fourth, there are different possible causes for respondents to become unemployed (voluntary or involuntary) or reasons to stop smoking, such as pregnancy or the price of cigarettes, which we could not take into account in our study. Last, we did not determine the exact duration of unemployment.

## CONCLUSIONS

Work loss was associated with a decrease in income, but not with an increase in psychological distress. Work loss, a decrease in income and an increase in psychological distress were not associated with quit intention, quit attempts and quit success, with one exception; an increase in psychological distress was associated with higher odds to make a quit attempt. Therefore, smokers who become unemployed with an associated decrease in income are not less likely to make quit attempts than smokers who are employed.
