# Supplementary material for: The role of income and psychological distress in the relationship between work loss and smoking cessation: Findings from three International Tobacco Control (ITC) Europe countries
Source: Tob Prev Cessat. 2019 Nov 19;5:42. doi: 10.18332/tpc/113092 (PMC7205135; doi:10.18332/tpc/113092)
Supplement: Supplementary file 1 [file TPC-5-42-s1.pdf]

Supplementary Table S1: Sample description

	Analysis sample (n=2712) %
**Sex**	
Male	51.8
**Age**	
15-24 years	14.5
25-39 years	33.1
40-54 years	37.3
55-65 years	15.0
**Education**	
Low	26.1
Moderate	37.5
High	36.4
**Country of residence**	
France	39.5
The Netherlands	40.4
Germany	20.2
**Living with a partner who smokes**	
Yes	33.8
**Number of friends who smoke**	
0	6.3
1	8.8
2	20.0
3	26.1
4	18.5
5	20.3
**Became unemployed between waves**	
Yes	8.5
**Income decreased between waves**	
Yes	23.8
**Psychological distress increased between waves**	
Yes	36.6
**Intention to quit in wave 2**	
Planning to quit within 6 months	23.6
Not planning to quit within 6 months	76.4
**Quit attempt made in wave 2**	
Yes	38.3
**Successfully quitted in wave 2 among those who tried**	
Yes	15.0

© 2019 Hummel K.
